# Development and Characterization of PLGA Nanoparticles Containing 17-DMAG, an Hsp90 Inhibitor

**DOI:** 10.3389/fchem.2021.644827

**Published:** 2021-05-13

**Authors:** Kercia P. Cruz, Beatriz F. C. Patricio, Vinícius C. Pires, Marina F. Amorim, Alan G. S. F. Pinho, Helenita C. Quadros, Diana A. S. Dantas, Marcelo H. C. Chaves, Fabio R. Formiga, Helvécio V. A. Rocha, Patrícia S. T. Veras

**Affiliations:** ^1^Laboratory of Parasite-Host Interaction and Epidemiology, Gonçalo Moniz Institute, Oswaldo Cruz Foundation (FIOCRUZ), Salvador, Brazil; ^2^Laboratory of Micro and Nanotechnology, Institute of Technology of Drugs (Farmanguinhos), Oswaldo Cruz Foundation (FIOCRUZ), Rio de Janeiro, Brazil; ^3^Laboratory of Tissue Engineering and Immunopharmacology, Gonçalo Moniz Institute, Oswaldo Cruz Foundation (FIOCRUZ), Salvador, Brazil; ^4^Department of Immunology, Aggeu Magalhães Institute (IAM), Oswaldo Cruz Foundation (FIOCRUZ), Recife, Brazil; ^5^Graduate Program in Applied Cellular and Molecular Biology, University of Pernambuco (UPE), Recife, Brazil; ^6^National Institute of Science and Technology of Tropical Diseases (INCT-DT), National Council for Scientific Research and Development (CNPq), Salvador, Brazil

**Keywords:** leishmaniasis, Hsp90, 17-DMAG, double emulsion, PLGA, nanoparticles

## Abstract

Leishmaniasis is a spectrum of neglected tropical diseases and its cutaneous form (CL) is characterized by papillary or ulcerated skin lesions that negatively impact patients' quality of life. Current CL treatments suffer limitations, such as severe side effects and high cost, making the search for new therapeutic alternatives an imperative. In this context, heat shock protein 90 (Hsp90) could present a novel therapeutic target, as evidence suggests that Hsp90 inhibitors, such as 17-Dimethylaminoethylamino-17-Demethoxygeldanamycin (17-DMAG), may represent promising chemotherapeutic agents against CL. As innovative input for formulation development of 17-DMAG, nano-based drug delivery systems could provide controlled release, targeting properties, and reduced drug toxicity. In this work, a double emulsion method was used to develop poly (lactic-co-glycolic acid) (PLGA) nanoparticles containing 17-DMAG. The nanoparticle was developed using two distinct protocols: Protocol 1 (P1) and Protocol 2 (P2), which differed concerning the organic solvent (acetone or dichloromethane, respectively) and procedure used to form double-emulsions (Ultra-Turrax® homogenization or sonication, respectively). The nanoparticles produced by P2 were comparatively smaller (305.5 vs. 489.0 nm) and more homogeneous polydispersion index (PdI) (0.129 vs. 0.33) than the ones made by P1. Afterward, the P2 was optimized and the best composition consisted of 2 mg of 17-DMAG, 100 mg of PLGA, 5% of polyethylene glycol (PEG 8000), 1.5 mL of the internal aqueous phase, 1% of polyvinyl alcohol (PVA), and 4 mL of the organic phase. Optimized P2 nanoparticles had a particle size of 297.2 nm (288.6–304.1) and encapsulation efficacy of 19.35% (15.42–42.18) by the supernatant method and 31.60% (19.9–48.79) by the filter/column method. Release kinetics performed at 37°C indicated that ~16% of the encapsulated 17-DMAG was released about to 72 h. In a separate set of experiments, a cell uptake assay employing confocal fluorescence microscopy revealed the internalization by macrophages of P2-optimized rhodamine B labeled nanoparticles at 30 min, 1, 2, 4, 6, 24, 48, and 72 h. Collectively, our results indicate the superior performance of P2 concerning the parameters used to assess nanoparticle development. Therefore, these findings warrant further research to evaluate optimized 17-DMAG-loaded nanoparticles (NP2-17-DMAG) for toxicity and antileishmanial effects *in vitro* and *in vivo*.

## Introduction

Constituting a severe public health problem throughout the world, the spectrum of leishmaniasis consists of neglected tropical diseases caused by parasite species of the genus *Leishmania*, 20 of which are capable of infecting humans (Masmoudi et al., 2013; Akhoundi et al., 2016; WHO, 2020a). Although endemic in 97 countries, the disease is mainly concentrated in Africa, Asia, and the Americas (WHO, 2020b). It is currently estimated that around 12 million people are infected worldwide, with an annual incidence of more than one million new cases per year; one billion of the world's population lives in areas at risk of infection (Akhoundi et al., 2016; WHO, 2020a).

Leishmaniasis can be divided into two primary clinical forms: cutaneous and visceral, with varying presentations depending on the species and virulence of the infecting parasite, as well as the type of host immune response (Kaye and Scott, 2011; Akhoundi et al., 2016; Oryan and Akbari, 2016; Srivastava et al., 2016; Veras and De Menezes, 2016; WHO, 2020a). Among the cutaneous presentations, mucocutaneous leishmaniasis (MCL), caused mainly by *L. aethiopica* in the Old World and *L. braziliensis* in the New World, is the most debilitating form, with destructive lesions occurring on the palate, lips and nasal septum (Akhoundi et al., 2016; Burza et al., 2018; WHO, 2020a). The most common form, localized cutaneous leishmaniasis (LCL), is caused by a variety of parasite species, including *L. major, L. tropica*, and *L. aethiopica* in the Old World, in addition to *L. braziliensis, L. guyanensis, L. amazonensis*, and *L. mexicana* in the New World (Kaye and Scott, 2011; Masmoudi et al., 2013; Burza et al., 2018; Meira and Gedamu, 2019). Despite not being fatal, LCL can affect patients' quality of life according to the evolution and spread of skin lesions, social stigmatization, psychological effects, and absenteeism (Carvalho et al., 1994; Scorza et al., 2017; Burza et al., 2018).

Currently, chemotherapy is the recommended treatment for patients diagnosed with leishmaniasis, mainly pentavalent antimonials and Amphotericin B in a free or liposomal-encapsulated form (Croft et al., 2006; Frézard et al., 2009; Seifert, 2011; Brasil, 2015; De Menezes et al., 2015; Andrade-Neto et al., 2018). Alternatively, other drugs, such as pentamidine and paromomycin, can also be applied in leishmaniasis treatment (Santos et al., 2008; De Menezes et al., 2015). These therapies present several limitations, including high cost, invasive route of administration, prolonged cycle and systemic side effects, e.g., weakness, myalgia, rigors/chills, hemolysis and fever, as well as instability at high temperatures in some formulations (Sundar et al., 2004; Frézard et al., 2009; Chávez-Fumagalli et al., 2015; De Menezes et al., 2015). Additionally, drug accumulation in the organs can lead to pancreatitis, nephrotoxicity, hepatotoxicity, myocarditis, and cardiotoxicity (Rath et al., 2003; Frézard et al., 2009; Seifert, 2011; Masmoudi et al., 2013; Chávez-Fumagalli et al., 2015; De Menezes et al., 2015). The only currently available non-invasive orally administered treatment for leishmaniasis, miltefosine, presents limitations including vomiting, diarrhea, kidney, and liver toxicity, as well as potential teratogenic effects and high cost in some regions (Rath et al., 2003; Seifert, 2011; Masmoudi et al., 2013; De Menezes et al., 2015; Andrade-Neto et al., 2018). This scenario highlights the need to discover new drugs that offer increased efficacy and less toxicity (De Menezes et al., 2015). So far, efforts to this end have focused on (i) increasing the safety and efficacy of treatments already in use; (ii) combined drug therapy via novel therapeutic protocols; (iii) the search for new therapeutic targets in parasites or host cells; (iv) repurposing drugs used to treat other diseases; (v) developing more effective delivery systems (Frézard et al., 2009; De Menezes et al., 2015; Andrade-Neto et al., 2018).

Heat Shock Protein 90 (Hsp90), a ubiquitous and highly conserved molecular chaperone, is responsible for performing the folding of other proteins, namely client proteins, subsequently preventing the post-translational formation of oligomeric complexes with incorrect, inactive and non-functional structures (Zhao and Houry, 2005; Erlejman et al., 2014). This chaperone has been described as a potential therapeutic target in treating cancer and infectious diseases caused by different parasite species, including those of the *Leishmania* genus (Solit and Chiosis, 2008; Pallavi et al., 2010; Roy et al., 2012; Whitesell and Lin, 2012; Schopf et al., 2017; Guswanto et al., 2018). Notably, in leishmaniasis, Hsp90 has been shown to aid in re-establishing the functional stability of proteins in response to environmental pressure, such as differences in pH and temperature, during parasite differentiation processes (Zilberstein and Shapira, 1994; Graefe et al., 2002; Solit and Chiosis, 2008; Pallavi et al., 2010; Roy et al., 2012; Hombach et al., 2014; Schopf et al., 2017).

Structurally, Hsp90 is comprised of three main domains: the intermediate central proteolytic domain, involved in the interface between Hsp90 and its client proteins; the C-terminal domain, which facilitates homodimerization; the N-terminal domain, responsible for interaction with and the hydrolysis of ATP (Pratt and Toft, 2003; Zhao and Houry, 2005; Brown et al., 2007). The family of benzoquinone ansamycins constitutes a class of Hsp90 inhibitors that compete with ATP for binding at the Hsp90 interaction site, thereby hindering chaperone activity (Zhao and Houry, 2005; Brown et al., 2007; Erlejman et al., 2014). Subsequently, truncated or malformed proteins become degraded by the ubiquitin-proteasome system (Chiosis et al., 2004; Xiao et al., 2006; Sidera and Patsavoudi, 2013). In parasites of the genus *Leishmania*, this inhibition leads to parasite death, evidencing the importance of Hsp90 in the maintenance of cellular homeostasis (Wiesgigl et al., 2001; Li et al., 2009; Roy et al., 2012; Hombach et al., 2013).

Research by our group previously demonstrated that geldanamycin (GA), 17-AAG, and 17-DMAG, Hsp90 inhibitors of benzoquinone ansamycin family, were capable of eliminating promastigote forms of *L. amazonensis* at concentrations determined as non-toxic for human monocyte lineage cells (THP-1) (Palma et al., 2019). It was also demonstrated that the treatment of *L. amazonensis-*infected macrophages with 17-AAG reduced the percentage of infected macrophages and numbers of intracellular parasites in a time- and dose-dependent manner at concentrations deemed non-toxic to host cells (Petersen et al., 2012). Furthermore, 17-AAG was found to control, both *in vitro* and *in vivo, L. braziliensis* infection in BALB/c mouse macrophages (Santos et al., 2014). This study demonstrates that 17-AAG reduces the size of ear lesions and parasitic load at the lesion site, but not in the draining lymph nodes of infected mice, resulting in infection relapse (Santos et al., 2014). To overcome this described limitation, it will be tested a water-soluble analog of 17-AAG, 17-DMAG (Egorin et al., 2002; Sausville, 2004; Whitesell and Lin, 2012). Because it is a water-soluble molecule and has better pharmacokinetics than 17-AAG, 17-DMAG can achieve lymph nodes of treated animals, eliminating the parasites on this site. To optimize a formulation containing-Hsp90 inhibitor for leishmaniasis treatment, we propose the encapsulation of 17-DMAG in a nanoparticle, which can have controlled release of the drug, prolonging its action with fewer administrations. This delivery system can also help prevent toxicity occurrences in future tests for *L. braziliensis* infection control.

Nanoparticle-based controlled release systems offer several advantages: improved safety, efficacy, target specificity (drug targeting), biocompatibility, bioavailability, biodegradability, and reduced toxicity in comparison to traditional drug delivery systems (Zhang et al., 2008; Formiga et al., 2009; Yildirimer et al., 2011; Lin, 2015; Utreja et al., 2020). By directing the active principle to specific tissues and releasing it gradually over time, the dose necessary to observe treatment efficacy becomes reduced, thereby contributing to a reduction in side effects (Yildirimer et al., 2011; Wolfram et al., 2015). Synthetic polymers such as poly(lactic-co-glycolic acid) (PLGA), polylactic acid (PLA) and polycaprolactone (PCL) are commonly used in drug delivery systems because they do not offer a risk of inducing an unwanted immune response (Formiga et al., 2009; Zhang and Zhang, 2017; Utreja et al., 2020). The main application of these systems consists of cases in which the free form of a drug presents limitations, such as shortened half-life, requiring the need for multiple applications, and inadequate target specificity, which can lead to the occurrence of a range of side effects (Zhang et al., 2008; Formiga et al., 2009). Thus, the present work aimed to produce polymeric nanoparticles (NPs) containing the Hsp90 inhibitor, 17-DMAG, and perform morphological and physical-chemical characterization. The results obtained herein will enable, in the future, an evaluation of *in vitro* and *in vivo* optimized 17-DMAG-loaded NPs (NP2-17-DMAG) as antileishmanial treatment.

## Materials and Methods

### Reagents and Chemicals

**Figure 1:**
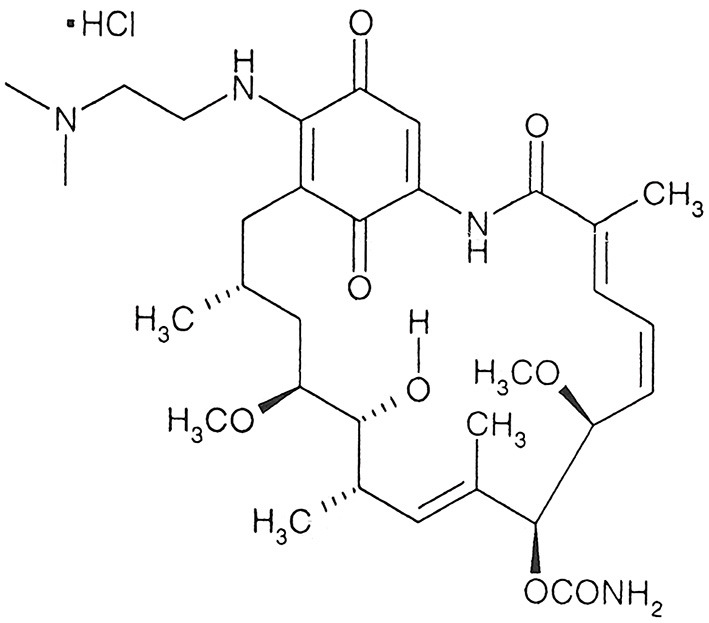
Chemical structure of 17-DMAG. Extracted from 17-DMAG datasheet (LC Laboratories).

The hydrochloride salt of alvespimycin, 17-DMAG (Figure 1), was purchased from LC Laboratories (Massachusetts, USA). Resomer® RG 503 H, Poly (D, L-lactide-co-glycolide) (50:50) (PLGA), Poly (vinyl alcohol) (87–90% hydrolyzed average mol wt 30,000–70,000, PVA) and poly(ethylene glycol) (PEG, MW = 8,000) were purchased from Sigma-Aldrich (Darmstadt, Germany). Alamar blue® was purchased from Invitrogen (Massachusetts, USA). Microcon-30 0.5ML microtubes were purchased from Merk Millipore (Darmstadt, Germany). A C18 HPLC column and C18 Supelguard Guard Cartridge were purchased from Sigma-Aldrich (Darmstadt, Germany).

### Preparations of PLGA NPs

Two different double emulsion protocols were used to prepare PLGA NPs. In the first protocol (P1), PLGA NPs containing 17-DMAG (NP1-17-DMAG) were prepared using a modified double emulsion/solvent evaporation technique (Salvador et al., 2015). Briefly, 50 mg of PLGA was dissolved in 5 mL of acetone, and 2 mg of 17-DMAG was dissolved in 5% PEG solution (250 mg in 5 mL of distilled water). The 17-DMAG solution was then added to the pre-cooled PLGA solution and emulsified using a Q700 sonicator (QSonica, Newtown, Connecticut, USA) at 6% amplitude for 2 min. Subsequently, 10 mL of PVA 1% w/v was added to the mixture. The recipient was then covered with aluminum foil and then homogenized at 10,000 rpm for 5 min (Ultra-turrax® T-25, IKA, Germany) to form a double emulsion. Next, 10 mL of 2% isopropyl alcohol was added. Finally, the suspension was magnetically stirred for 30 min with a subsequent solvent evaporation step using a rotary evaporator (IKA) for 1 h at 56°C under 250 mBar.

In the second double emulsion protocol (P2), PLGA NPs (NP2-17-DMAG) were also produced using another double emulsion/solvent evaporation technique (Mainardes et al., 2009). Initially, 100 mg of PLGA was dissolved in 4 mL of dichloromethane. Another solution containing 2 mg of 17-DMAG dissolved in 1.5 mL of PEG 5% w/v was then added to the pre-cooled PLGA solution to form a single emulsion using a Q700 sonicator (QSonica, Newtown, Connecticut, USA) at 40% amplitude for 1 min. To form the double emulsion, the single emulsion was incorporated in 10 mL of PVA 1% w/v, then sonicated. Finally, 30 mL of PVA 1% w/v was added to the recipient, covered with aluminum foil and magnetically stirred for 30 min. The solvent extraction step was performed using a rotary evaporator (IKA) for 1 h at 40°C under 200 mBar.

Following each nanoparticle protocol preparation, suspensions were washed in distilled water three times at 39,800 × g for 15 min at 4°C (T-865, Thermo Fisher Scientific, Massachusetts, USA). Samples were frozen at −80°C, then lyophilized for 24 h at −48°C under 0.050 mBar (FreeZone 2.5 Liter Benchtop, Labconco, USA) and subsequently stored at 4°C. For blank nanoparticle preparation (NP1-Ø and NP2-Ø), these same protocols were followed in the absence of 17-DMAG.

To evaluate the influence of PLGA and PEG concentration on the encapsulation efficiency (%EE) of 17-DMAG and the size of the produced NPs, P2 was performed as described above using 100 or 200 mg of PLGA and 2.5% w/v or 5% w/v of PEG.

### PLGA NPs Characterization

#### Dynamic Light Scattering

DLS was used to measure the particle size, polydispersion index (PdI) and zeta potential (ZP) of the obtained NPs. After washing, NP-17-DMAG or NP-Ø produced by the P1 or P2 double-emulsion protocols were resuspended in 5 mL of distilled water and diluted 1:125 in distilled water. DLS analysis was performed in triplicate using a ZetaSizer Nano ZS90 (Malvern Panalytical, UK) at 25°C.

#### Transmission Electron Microscopy

For imaging and size confirmation, NPs were washed as described in item 2.2 and analyzed by TEM. Aliquots of NP-17-DMAG or NP-Ø produced by the P1 or P2 double-emulsion protocols were diluted 1:10 in distilled water, then 10 μL of each sample was placed on a formvar film-coated grid and stained with 2% uranyl acetate for 2 min. TEM analysis was performed using a JEM-1230 transmission electron microscope (JEOL LTD, Japan).

#### Scanning Electron Microscopy

SEM was used to examine the shape and surface morphology of the NP-17-DMAG or NP-Ø produced by the P1 or P2 double-emulsion protocols. For SEM analysis, lyophilized NPs (1 mg) were placed on an adhesive stub and coated with gold-palladium under vacuum using an ion coater. All samples were analyzed and photographed at 15 kV using a JSM-6390LV microscope (JEOL LTD, Japan).

### HPLC for 17-DMAG Quantification

To quantify 17-DMAG, HPLC was performed using a C18 HPLC column and C18 Supelguard Guard Cartridge following manufacturer protocols. First, the mobile phase was prepared using HPLC grade acetonitrile (27%), HPLC grade methanol (27%), ultrapure water (46%), and trifluoroacetic acid (0.05%). In parallel, 17-DMAG was diluted in distilled water at an initial concentration of 500 μg/mL, then diluted from 50 to 1 μg/mL in the mobile phase to construct concentration curves in triplicate. Each sample was analyzed at a 2D wavelength of 254 nm for 8 min at 25°C. The mobile phase flow rate was 1 mL/min, with 10 μL of each sample injected. The observed retention time for 17-DMAG under these conditions was ~3.8 min. Nanoparticle concentrations of 17-DMAG were calculated using a free compound curve (Empower software, version 3).

### Encapsulation Efficiency (%EE) of 17-DMAG in NPs

Encapsulation efficiency determination was performed using two indirect methods: filter/column or supernatant. After the solvent was evaporated using the filter/column method, 500 μL of nanoparticle suspension was centrifuged in a 1.5 mL microtube with a Microcon 30 filter under 14,000 × g for 1 h at 4°C. As the free drug fraction (F) in each sample flowed through the filter, NPs were retained. In parallel, absolute ethanol was added to another 500 μL aliquot of total nanoparticle suspension at a proportion of 1:1 to determine the total amount of 17-DMAG. After centrifugation at 6,200 × g for 15 min at 4°C, the supernatant was collected and the total quantity of 17-DMAG (T) was measured. %EE was evaluated by HPLC following the formula:

$$\begin{array}{l} \%EE=\frac{\left(T-F\right)}{T}\times \;100, \end{array}$$

where T corresponds to the total mass of the drug in the sample, whether encapsulated or not, and F corresponds to the non-encapsulated fraction (free fraction).

For the supernatant method, the supernatants obtained from three washes of the produced NPs were collected and then diluted at 1:5 in the mobile phase. HPLC then evaluated the %EE according to the formula:

$$\begin{array}{l} \%EE=\frac{S}{T}\times \;100, \end{array}$$

where S corresponds to the total mass of 17-DMAG in the supernatants and T is the total mass of the drug added for encapsulation.

### Release of 17-DMAG From NP2-17-DMAG *in vitro*

The release of 17-DMAG from NP2-17-DMAG was assessed *in vitro* using a modified method to determine the release kinetic profile (Quadros et al., 2020). Briefly, 2 mg of NP2-17-DMAG were placed into 1.5 mL polypropylene microcentrifuge tubes and resuspended in 1 mL of Dulbecco's modified Eagle's medium (DMEM) (Gibco), supplemented with 20 mM of HEPES (Sigma), 42.14 mM of sodium bicarbonate (Sigma), 10% of inactivated fetal bovine serum (Gibco), 2 mM of glutamine (Sigma), and 20 μg/mL of ciprofloxacin (Isofarma, Precabura, CE, BR) (complete DMEM medium). Next, the sealed tubes were placed in a rotating shaker and maintained at 37°C for 72 h. At each specific time point (1, 3, 6, 12, 24, 48, and 72 h), the sample tubes were removed from the incubator and centrifuged at 21,000 × g for 15 min at 4°C. The supernatant was then collected, frozen and immediately replaced with an equal volume of fresh release medium. To determine the 17-DMAG concentration, collected supernatants were diluted in the mobile phase and quantified using HPLC Shimadzu LC20-A (São Paulo, Brazil). All assays were performed in triplicate.

### Animal Manipulation and Ethics Statement

BALB/c mice, male or female, aged 6–12 weeks, were provided by the Gonçalo Moniz Institute (IGM/FIOCRUZ) Animal Care Facility. The animals were maintained under pathogen-free conditions, with food and water *ad libitum*. All procedures involving animals were conducted under the International Guiding Principles for Biomedical Research Involving Animals. The Institutional Review Board approved this study's experimental design (CEUA protocol no. 007/2020) of the Gonçalo Moniz Institute, Bahia-Brazil (IGM–FIOCRUZ/BA).

### Obtainment of Bone Marrow-Derived Macrophages From BALB/c Mice

BALB/c mice were euthanized using thiopental intraperitoneal injection (50 mg/kg). Mouse femurs and tibias were aseptically removed and kept in cold 0.9% NaCl solution containing 0.01 mg/mL of ciprofloxacin. In a sterile environment, bone extremities were removed and marrow cells were extracted by washing the bone cavity with Roswell Park Memorial Institute (RPMI) 1640 medium (GIBCO) supplemented with 20 mM of HEPES (SIGMA), 23 mM of sodium bicarbonate (SIGMA), 10% of inactivated fetal bovine serum (Gibco), 2 mM of Glutamine (Sigma) and 20 μg/mL of ciprofloxacin (Isofarma, Precabura, CE, BR) (complete RPMI medium). Extracted marrow cells were centrifuged at 300 × g at 4°C for 10 min, then resuspended and cultivated in Petri dishes (three plates per animal) containing 10 mL of complete RPMI medium with 30% supernatant from L929 cell culture containing granulocyte macrophage colony-stimulating factor (GM-CSF). The dishes were cultivated at 37°C under 5% CO_2_ and 95% humidity for 24 h, after which the supernatant was transferred to new plates. After 72 h, an additional 5 mL of complete RPMI medium containing 30% L929 supernatant was added to each culture to re-stimulate cells for differentiation.

On the 7th day, BMDM were recovered from bacterial Petri dishes using 5 mL of 1 mM EDTA solution (pH 8.0) for 5 min at 37°C. Cells were centrifuged at 300 × g for 10 min at 4°C, then resuspended in 1 mL of complete DMEM medium and counted in a Neubauer chamber.

### Uptake of Fluorescent NPs by BMDM *in vitro*

NPs containing rhodamine B (Sigma) were produced using the P2 protocol (item 2.2). BMDM were obtained as described above and plated at 10^5^ cells per well in 1 mL of complete DMEM medium on 24-well plates containing glass coverslips. For the *in vitro* uptake assay, rhodamine B-containing NPs were lyophilized and then incubated with BMDM for 30 min, 1, 2, 4, 6, 24, 48, and 72 h. Wells were washed at each time point, and cells were fixed with 4% paraformaldehyde (PFA) for 15 min at room temperature. Finally, coverslips were mounted on slides using ProLong Gold antifade with DAPI® (Invitrogen, Darmstadt, Germany). Images were obtained by confocal fluorescence microscopy using a Leica SP8 device (Leica Microsystems, Mannheim, Germany).

### Statistical Analysis

Graphs were constructed and statistical analyses were performed using GraphPad Prism version 5.01 (GraphPad Software Inc). The Kolmogorov-Smirnov test was employed to verify normality. For data with Gaussian distribution, Student's *t*-test or one-way ANOVA were used to compare between two groups or among three or more groups, respectively, followed by Tukey's post-test. For non-gaussian distributions, the Mann-Whitney U test was applied for comparisons between two groups, while Kruskal-Wallis was used to compare three or more groups. Differences were considered statistically significant when *p* < 0.05.

## Results

### DLS Characterization and %EE of NPs Produced by P1 or P2 Double Emulsion Protocols

The NP1-17-DMAG had a larger average size, size variation (PdI), and ZP than the NP2-17-DMAG (Table 1). No differences were detected in %EE values regardless of the quantitation method (supernatant or filter/column) used to determine the amount of 17-DMAG encapsulated in NP1-17-DMAG or NP2-17-DMAG (Table 1).

**Table 1:** Particle size (Size), polydispersion index (PdI), zeta potential (ZP) and encapsulation efficiency (%EE) by supernatant of filter/column methods of NPs produced by P1 or P2 double emulsion protocols.

**Protocol**	**Size (nm)**	**PdI**	**ZP (mV)**	**%EE\break (supernatant)**	**%EE (filter\break/column)**
P1	489	0.33	−34.4	14.9%	14.02%
P2	305.5	0.129	−28.7	15.04%	17%

### Morphological Characterization of NP1 and NP2

**Figure 2:**
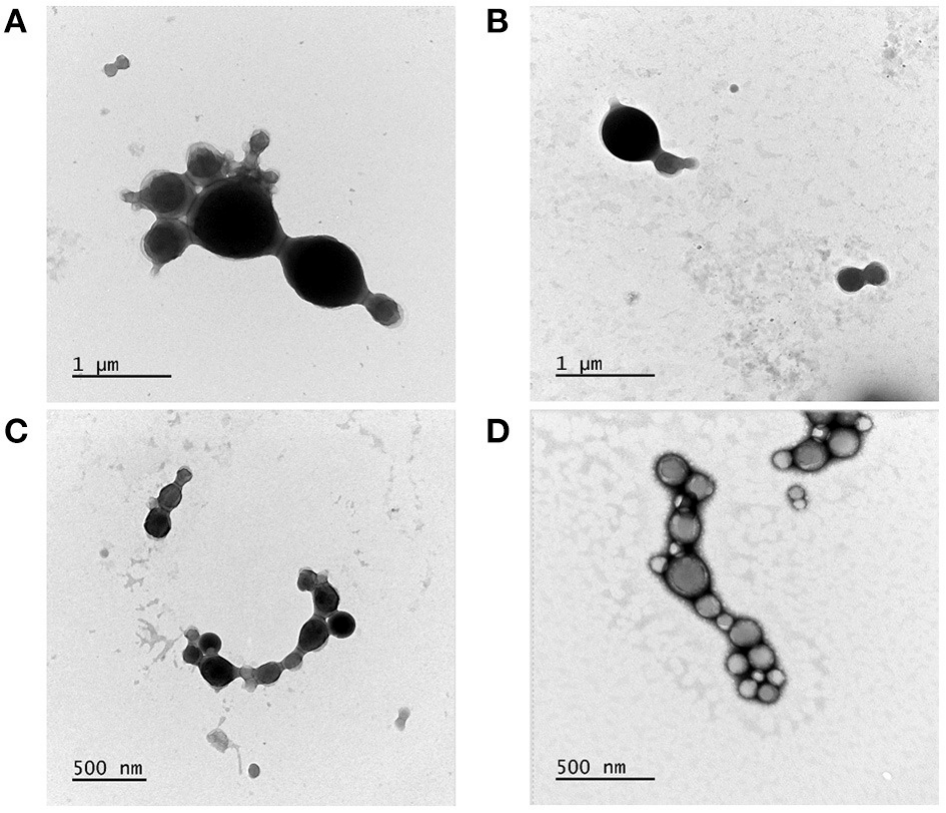
Morphological characterization of NP1 and NP2 by TEM. Transmission electron microscopy images of NP-Ø **(A,C)** and NP-17-DMAG **(B,D)** produced by P1 **(A,B)** or P2 **(C,D)** double emulsion protocols. 10 μL of each sample was contrasted with 2% uranyl acetate for 2 min in a formvar grid. Bars represent size references in μm or nm.

Consistent with the obtained PdI values, electron microscopy analysis revealed a more significant size variation in NP1 (Figures 2A,B) compared to NP2 (Figures 2C,D). Both protocols produced spherical, regular-shaped NPs (Figure 2). No morphological differences were observed between NP-Ø and NP-17-DMAG.

**Figure 3:**
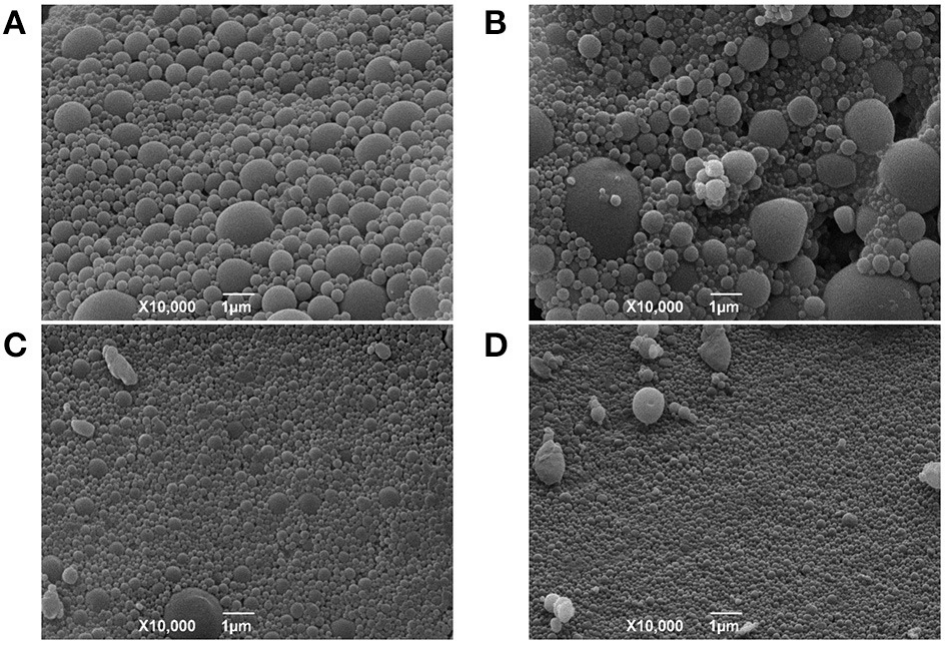
SEM evaluations of NP1 and NP2. NP-Ø **(A,C)** or NP-17-DMAG **(B,D)** were produced by P1 **(A,B)** or P2 **(C,D)** double emulsion protocols. The produced NPs were frozen at −80°C and lyophilized for 24 h at −48°C under 0.050 mbar. Approximately 1 mg of each sample was vacuum-coated with gold-palladium using an ion coater and analyzed by SEM. All samples were analyzed and photographed at 15 kV. Bars represent 1 μm.

SEM morphological analysis confirmed the spherical shape and smooth surface of the NPs produced by both double emulsion protocols (Figure 3). Consistent with DLS and TEM results, SEM analysis also revealed that NP1-Ø and NP1-17-DMAG exhibited more considerable size variation (Figures 3A,B) compared to NP2-Ø and NP2-17-DMAG (Figures 3C,D). Again, no morphological differences were observed between NP-Ø (Figures 3A,C) and NP-17-DMAG (Figures 3B,D) regardless of the protocol used.

### P2 Optimization Protocol

**Figure 4:**
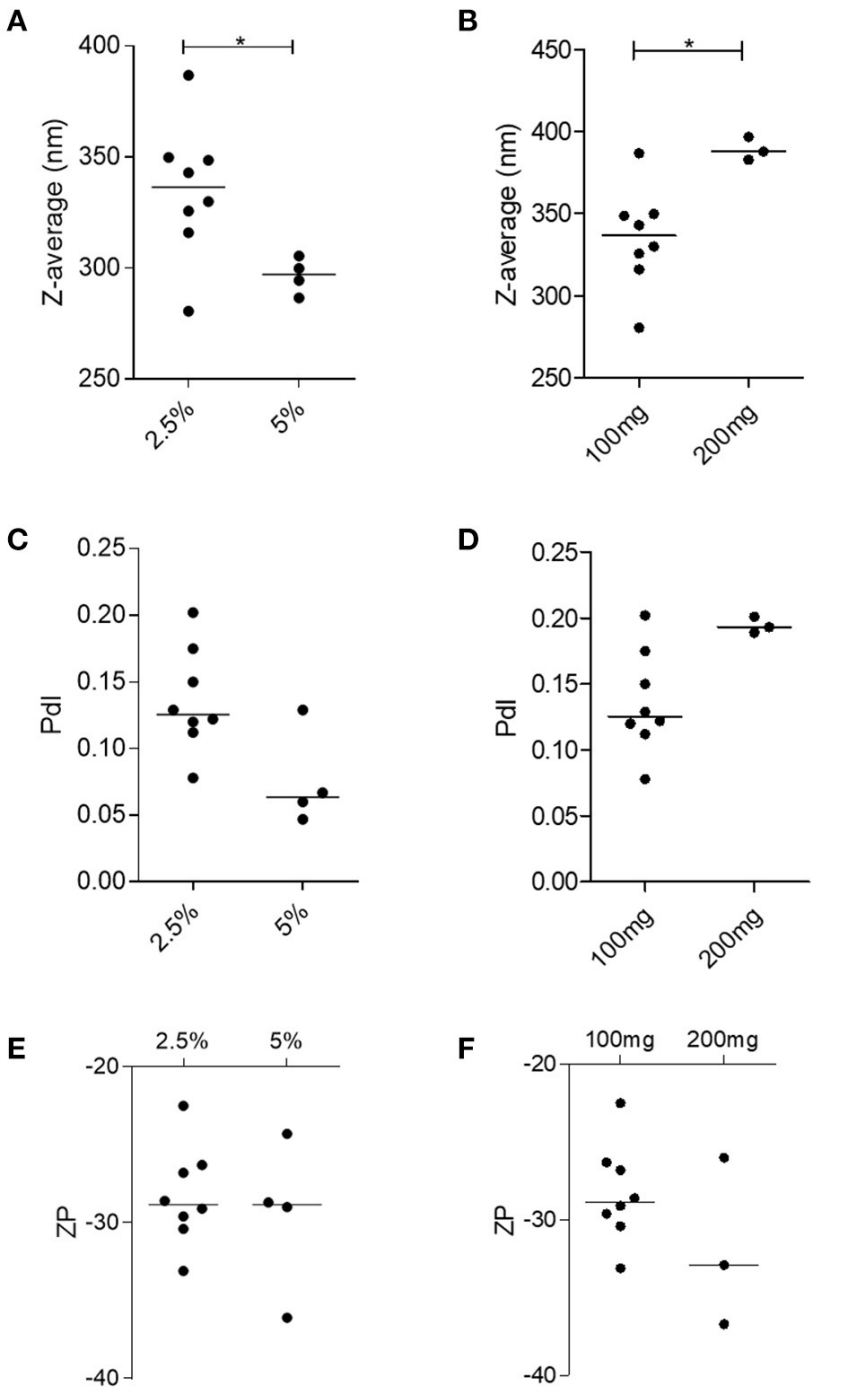
Evaluation of the influence of amount of PLGA and PEG concentration on nanoparticle physiochemical features in NP2-17-DMAG. DLS evaluated the effect of varying amounts of PEG **(A,C,E)** and PLGA **(B,D,F)** on the size **(A,B)**, PdI **(C,D)**, and ZP **(E,F)** of NP2-17-DMAG. Each point represents an individual experiment. Mann-Whitney test, *p < 0.05.

As NP2-17-DMAG exhibited superior physical-chemical and morphological characteristics compared to NP1-17-DMAG, we employed P2 to optimize the 17-DMAG encapsulation process with some variations. NPs produced using 5% PEG presented a smaller median size of 297.2 nm (Q1: 288.6; Q2:304.1) compared to those made using 2.5% PEG (median size: 336.5 nm; Q1: 318.4; Q2: 349.6) (Figure 4A). Similarly, NPs produced containing 100 mg of PLGA presented a smaller median size of 336.5 nm (Q1: 318.4; Q2: 349.6) in comparison to those containing 200 mg of PLGA (median size: 387.8 nm; Q1: 382.8; Q2: 396.7) (Figure 4B). No differences were detected concerning PdI and ZP values among NPs prepared using different PEG concentrations (Figures 4C,E), nor different PLGA masses (Figures 4D,F).

**Figure 5:**
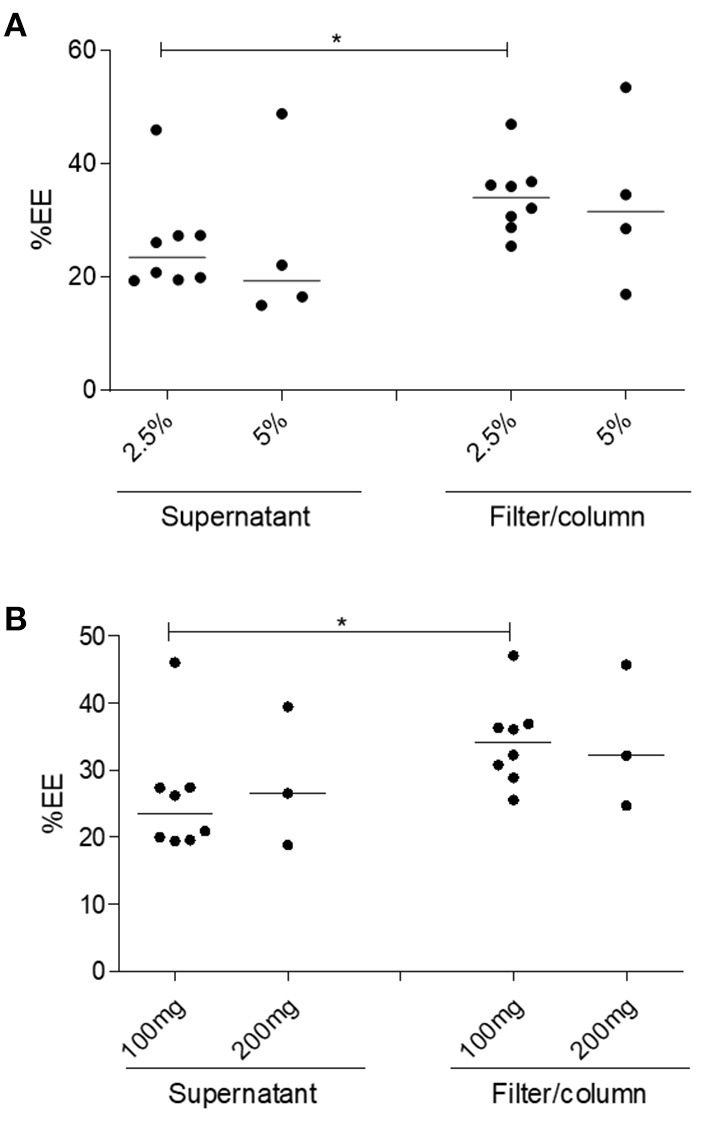
Evaluation of the influence of PLGA and PEG concentration on the %EE of 17-DMAG in NP2-17-DMAG. The effect of varying amounts of PEG **(A)** and PLGA **(B)** on the %EE of 17-DMAG in NP2-17-DMAG was determined by filter/column or supernatant analysis methods as described in section Material and Methods. Measurements were performed using HPLC. Each point represents an individual experiment. Mann-Whitney test, *p < 0.05.

Similar %EE values were found for NP2-17-DMAG regardless of the amount of PEG used (2.5 or 5%) (Figure 5A). Median %EE values for 2.5 and 5% of PEG were 23.51% (Q1: 19.66; Q2: 27.38) and 19.35% (Q1: 15.42; Q2: 42.18), respectively, using the supernatant analysis method, vs. 34.12% (Q1: 29.31; Q2: 36.73) and 31.60% (Q1: 19.90; Q2: 48.79) using the filter/column method (Figure 5A). Furthermore, similar %EE results were seen in NPs produced with different amounts of PLGA (Figure 5B) using the supernatant quantitative analysis method, with respective median values of 23.51% (Q1: 19.66; Q2: 27.38) and 26.48% (Q1: 18.78; Q2: 39.39) for 100 and 200 mg of PLGA, respectively. The filter/column method yielded median %EE values of 34.12% (Q1: 29.31; Q2: 36.73) for 100 mg and 32.12% (Q1: 24.66; Q2: 44.63) for 200 mg of this polymer. It is worth noting that, in comparison to the supernatant analysis method, higher %EE results were obtained using the quantitative filter/column method regardless of NP2 protocol modifications (Figure 5).

### *In vitro* Release of 17-DMAG From NP2-17-DMAG

**Figure 6:**
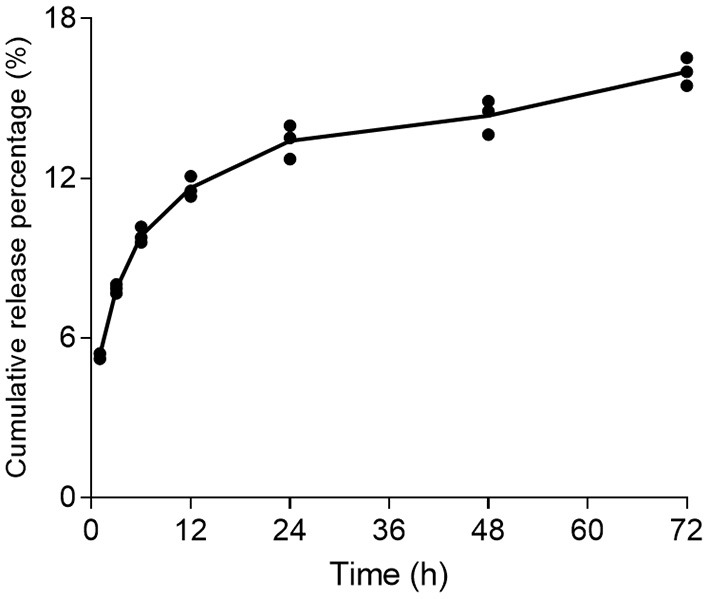
Kinetics of 17-DMAG release from NP2-17-DMAG. NP2-17-DMAG were lyophilized and incubated in complete DMEM medium under rotary agitation at 37°C. After 1, 3, 6, 12, 24, 48 or 72 h, samples were centrifuged, the supernatant was collected, and the medium was replaced. The release of 17-DMAG was quantified in supernatants using HPLC. Mean, *n* = 3.

At 1, 3, 6, 12, 24, 48, and 72 h of incubation, 5.36, 7.86, 9.85, 11.64, 13.41, 14.36, and 16% of 17-DMAG were cumulatively released from NP2-17-DMAG (Figure 6) *in vitro*, i.e., the amount of 17-DMAG was observed to continuously increase in complete DMEM medium for 72 h (Figure 6).

### Uptake of Fluorescent NPs by BMDM *in vitro*

**Figure 7:**
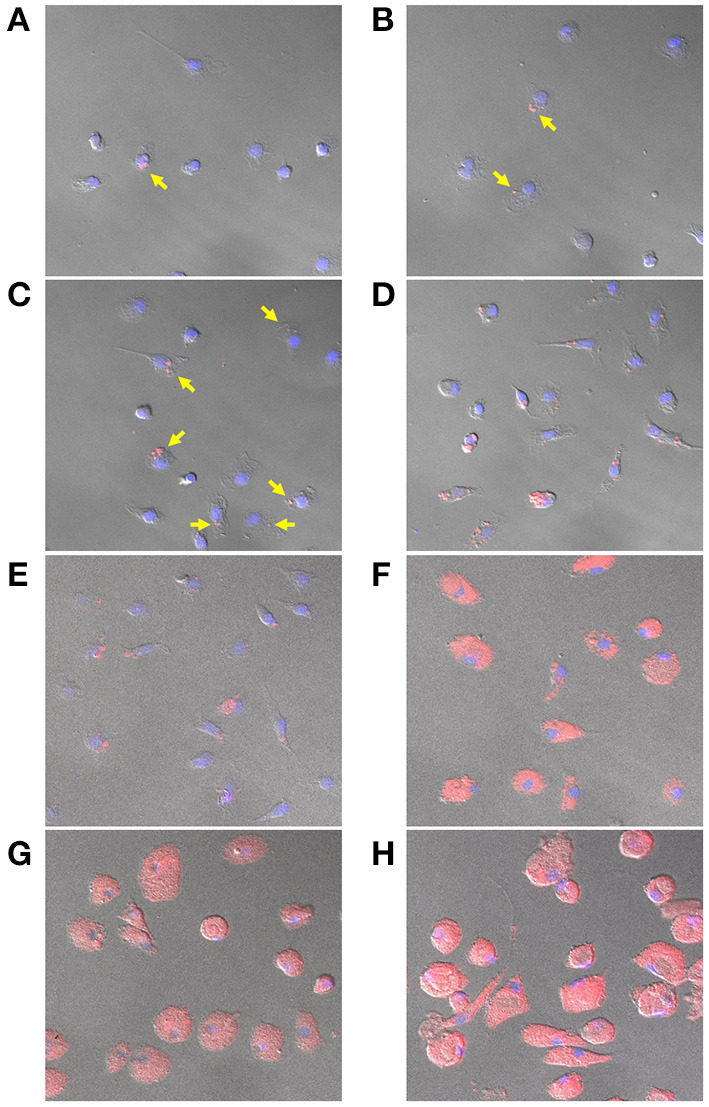
Uptake of NP2-rhodamine by BMDM. BMDM were plated at 10^5^ cells per well and incubated with NP2-rhodamine for 30 min **(A)**, 1 h **(B)**, 2 h **(C)**, 4 h **(D)**, 6 h **(E)**, 24 h **(F)**, 48 h **(G)** or 72 h **(H)**. Following incubation, cells were fixed with 4% PFA and slides were mounted with DAPI. Cells were analyzed by confocal fluorescence microscopy (Leica SP8). Blue = DAPI; Red = rhodamine. Arrows indicate internalization of NP2-rhodamine by macrophages at early timepoints.

The rhodamine-encapsulated NPs produced by P2 (NP2-rhodamine) presented similar size, shape, and appearance (smooth surface) as the morphological characteristics of NPs produced with or without 17-DMAG (data not shown). The uptake of NP2-rhodamine by BMDM was observed at an early incubation time of 30 min, 1, 2, 4, and 6 h (Figures 7A–E, respectively). After 24, 48 or 72 h (Figures 7F–H, respectively), greater numbers of NPs were observed in the cytoplasm of BMDM, indicating continued uptake by these cells.

## Discussion

The present work encapsulated 17-DMAG in PLGA NPs and investigated the resulting physical-chemical, morphological, and biological parameters. To standardize NP production, two double emulsion-solvent evaporation protocols (P1 and P2) were used (Astete and Sabliov, 2006; Mainardes et al., 2009). Analyses by DLS, TEM and SEM showed that NP1 presented larger sizes and higher PdI than NP2, which agrees with a previous report (Astete and Sabliov, 2006). We found that modifications in the PLGA-NP production protocol, such as the nature of the organic solvent used or the emulsion method, altered the characteristics of the produced NP. The larger sizes presented by NP1 may be due to laminar flow under stirring, such as that produced by the Ultra-Turrax® dispersing device. Accordingly, monodispersed drops may form in the emulsion, increasing the size of the produced NP, which is not observed under sonication (Astete and Sabliov, 2006). Concerning ZP, a measurement of the electrical behavior of NPs, both protocols produced NPs with values around −30 mV, indicating stability with minimal aggregate formation (Formiga et al., 2009). Furthermore, the observed similarity between %EE values in each protocol can be justified by using the same polymer and surfactant (PEG), as these parameters significantly influence the entrapment of hydrophilic drugs (Astete and Sabliov, 2006).

The morphological characterization of NP1 and NP2 by TEM and SEM confirmed differences in size and PdI between the two protocols, revealing a similarly regular, spherical shape and smooth surface appearance. These aspects are essential as the morphological characteristics of NPs can predict possible interactions with living cells and uptake, intracellular localization and toxicity (Shang et al., 2014). Our results agree with previous studies (Cohen-Sela et al., 2009; McCall and Sirianni, 2013; Garbuzenko et al., 2014), demonstrating that PLGA particles produced by double emulsion presented spherical and regular shapes with smooth surfaces, regardless of modifications performed in the protocol. Following DLS, TEM and SEM characterization, P2 was selected as the better protocol for NP-17-DMAG production.

We then evaluated the effects of modifications to P2: NP2-17-DMAG produced with 100 mg of PLGA presented smaller sizes than 200 mg. This result stands in accordance with other authors who observed that increasing concentrations of PLGA generated larger particles (Astete and Sabliov, 2006; Rizkalla et al., 2006; Hernández-Giottonini et al., 2020). This has been observed to occur due to increased viscosity in the primary emulsion (w/o), which results in a less-efficient particle size reduction during the double emulsification process (w/o/w) (Iqbal et al., 2015). Similarly, using 5% PEG compared to 2.5% resulted in a smaller NP2-17-DMAG size. The decreased size was also observed at higher PEG concentrations in other studies (Zambaux et al., 1998; Rizkalla et al., 2006; Iqbal et al., 2015; Urbaniak and Musiał, 2019; Hernández-Giottonini et al., 2020). Higher surfactant concentrations reduce surface tension and promote particle division during the homogenization process, thus decreasing the size of the particles formed (Keum et al., 2011; Fonte et al., 2012). This phenomenon is maintained until a saturation point is reached (Keum et al., 2011; Fonte et al., 2012; Iqbal et al., 2015; Urbaniak and Musiał, 2019).

Encapsulation efficiency (%EE) was measured indirectly in this study. Two methods, filter/column or supernatant, were employed to separate loaded NPs and the non-encapsulated drug. We identified higher %EE values using filter/column than the supernatant, which may be attributable to superior free drug separation by this first method. This finding is consistent with studies on nanoparticle purification efficiency (Akbulut et al., 2012; Robertson et al., 2016), demonstrating that nanoparticle separation by supernatant depends on nanoparticle size and shape, the molecular weight of the polymer, dispersion medium density, speed, and centrifugation time. The filter/column method has commonly been considered a more robust, more straightforward and efficient purification process (Robertson et al., 2016; Shah et al., 2020).

After characterizing NP2-17-DMAG, we evaluated the kinetics of drug release. We found that NP2-17-DMAG exhibited two release phases: a preliminary rapid release of 17-DMAG lasting up to 24 h, followed by a slow and sustained release from 24 to 72 h. This data is similar to results obtained by Rietscher et al. (2016) and Rafiei and Azita (2017), who analyzed the release of different compounds in PLGA or PLGA-PEG NPs. PLGA NPs containing paromomycin also presented a similar release profile (Afzal et al., 2019), which was expected for polymeric NPs encapsulating hydrophilic molecules. At the initial timepoints evaluated, the drug is rapidly released due to adsorbed molecules on the surface of NPs (Hirenkumar and Steven, 2012; Kapoor et al., 2015; Rietscher et al., 2016; Mir et al., 2017; Rafiei and Azita, 2017). In contrast, at subsequent time points, a sustained and slower release occurs due to gradual degradation of the polymeric matrix and slowly release of the drug contained there (Hirenkumar and Steven, 2012; Kapoor et al., 2015; Rietscher et al., 2016; Mir et al., 2017; Rafiei and Azita, 2017).

Nanoparticle uptake was evaluated through the incubation of BMDM with NP2-rhodamine. Fluorescence microscopy analysis revealed the internalization of NPs by macrophages beginning at early times of contact with NP2-rhodamine. At later timepoints, these particles continued to be internalized and accumulation was observed in these cells' cytoplasm. These results are in agreement with other studies of professional phagocytes, including RAW 264 and J774 macrophage cell-lines and primary resident and inflammatory macrophages, which have been shown to internalize NPs at early timepoints, such as at 30 min of incubation (Cohen-Sela et al., 2009; Nicolete et al., 2011; Petersen et al., 2018; Couto et al., 2020; Van Hees et al., 2020).

The present study revealed that PLGA NPs containing 17-DMAG prepared using a double emulsion protocol presents physical-chemical, morphological, and biological characteristics conducive to CL's treatment. Additional studies will be carried out to investigate biological and immunological effects of NP2-17-DMAG in *L. braziliensis* infection control both *in vitro* and *in vivo* in a future manuscript.

## Data Availability Statement

The raw data supporting the conclusions of this article will be made available by the authors, without undue reservation.

## Ethics Statement

The animal study was reviewed and approved by BALB/c mice, male or female, aged 6-12 weeks, were provided by the Gonçalo Moniz Institute (IGM/FIOCRUZ) Animal Care Facility. The animals were maintained under pathogen-free conditions, with food and water *ad libitum*. All procedures involving animals were conducted under the International Guiding Principles for Biomedical Research Involving Animals. The Institutional Review Board approved this study's experimental design (CEUA protocol no. 007/2020) of the Gonçalo Moniz Institute, Fiocruz, Salvador, Brazil.

## Author Contributions

KC, BP, VP, FF, HR, and PV conceptualized and designed the experiments and analyzed and validated the data analysis. KC, BP, VP, MA, AP, DD, and MC performed the experiments. KC, VP, MA, AP, HQ, and PV wrote the paper. KC, BP, HR, FF, and PV reviewed and edited this paper. All authors contributed to the article and approved the submitted version.

## Conflict of Interest

The authors declare that the research was conducted in the absence of any commercial or financial relationships that could be construed as a potential conflict of interest.
